# Hydrolyzed Proteins and Vegetable Peptides: Anti-Inflammatory Mechanisms in Obesity and Potential Therapeutic Targets

**DOI:** 10.3390/nu14030690

**Published:** 2022-02-06

**Authors:** Amanda Fernandes de Medeiros, Jaluza Luana Carvalho de Queiroz, Bruna Leal Lima Maciel, Ana Heloneida de Araújo Morais

**Affiliations:** 1 Postgraduate Biochemistry and Biology Molecular Program, Biosciences Center, Federal University of Rio Grande do Norte, Natal 59078-970, RN, Brazil; amanda-nut@hotmail.com (A.F.d.M.); luh_nutri@hotmail.com (J.L.C.d.Q.); 2 Department of Nutrition, Center for Health Sciences, Federal University of Rio Grande do Norte, Natal 59078-970, RN, Brazil; brunalimamaciel@gmail.com; 3 Postgraduate Nutrition Program, Center for Health Sciences, Federal University of Rio Grande do Norte, Natal 59078-970, RN, Brazil

**Keywords:** amino acids, peptides and proteins, anti-inflammatory agents, anti-obesity agents, inflammation mediators

## Abstract

Chronic low-grade inflammation is present in overweight and obesity, causing changes in several metabolic pathways. It impairs systemic functioning and positively feeds back the accumulation of more adipose tissue. Studies with hydrolyzed proteins and plant peptides have demonstrated a potential anti-inflammatory and immunomodulatory effect of these peptides. However, it is challenging and necessary to explore the mechanism of action of such molecules because understanding their effects depends on their structural characterizations. Furthermore, the structure might also give insights into safety, efficacy and efficiency, with a view of a possible health application. Thus, the present narrative review aimed to discuss the mechanisms of action of hydrolyzed proteins and plant peptides as anti-inflammatory agents in obesity. Keywords and related terms were inserted into databases for the search. Based on the studies evaluated, these biomolecules act by different pathways, favoring the reduction of inflammatory cytokines and adipokines and the polarization of macrophages to the M2 phenotype. Finally, as a future perspective, bioinformatics is suggested as a tool to help understand and better use these molecules considering their applicability in pre-clinical and clinical studies.

## 1. Introduction

Obesity and overweight represent a serious global public health problem, and obesity is considered a pandemic [1], with considerable economic impacts [2]. According to the World Health Organization (WHO) data, in adults, obesity has almost tripled since 1975 and more than quadrupled in children and adolescents [3,4].

Overweight and obesity are, as defined by the WHO, “abnormal or excessive accumulation of fat that can harm health” [3]. The excess of adipose tissue, especially the visceral, progressively favors the emergence of insulin resistance, inflammation, lipotoxicity, mitochondrial dysfunction and alteration in protein synthesis, and these disorders can result in diseases such as type 2 diabetes mellitus (T2DM), dyslipidemias, cardiovascular diseases (CVDs), hepatic steatosis and autoimmune diseases [5,6,7,8].

In overweight, there is a low-grade systemic chronic inflammatory response, which favors the establishment of obesity, generating a “vicious cycle” that positively feeds back the accumulation of more adipose tissue and activates immune cells, releasing cytokine and adipokines [6,8,9,10,11].

The first line of action for the treatment of obesity is changing habits for a healthy diet and physical activity practice. However, individuals commonly present difficulty and/or low access to primary treatment. According to the need of each case, it is necessary to use medications, which have adverse effects and side effects. Thus, studying potential molecules, such as plant peptides, can be a strategy for treatment with adjuvants [12,13].

Several studies explore the action of anti-inflammatory agents [13,14,15,16], such as proteins of vegetable origin [14,15] and bioactive peptides [17].

Bioactive peptides and hydrolyzed proteins, different from integrative proteins, commonly present low molecular weight structures, favoring digestibility and bioavailability [18]. In addition, peptides have several physical–chemical properties that involve solubility, the possibility of emulsification, affinity with lipids and foaming, depending on the amino acid constitution, structure and size. This versatility attracts the pharmaceutical and food industry to develop foods with added functional value [19].

Thus, increasingly, the industry has invested significantly in research in this area. However, standardization of the process of purification and characterization of bioactive peptides is necessary in regard to the following steps: (1) extraction, fractionation and direct protein isolation from the source; (2) the characterization of the proteins of interest; (3) hydrolysis, which, at the end of the digestion process, will result in a pool of peptides; (4) purification, characterization and sequencing of each peptide released from hydrolysis; (5) bioactivity tests of peptides, registering all relevant data in the bioinformatics database, (6) most promising peptides can be synthesized for in vitro, pre-clinical and clinical trials. All the processes and also the synthesis have a high cost [19,20,21].

Due to the lengthy process and high cost for purification and characterization, it is of paramount importance to compile data of hydrolyzed proteins and peptides from plant sources to understand the mechanism of action of these molecules in the search for candidates with potential anti-inflammatory and immunomodulatory effects.

Although proteins of plant origin are widely studied, the universe of the effects of bioactive peptides derived from vegetables is still unknown, and it is necessary to explore them also considering mechanistic aspects. Therefore, based on the importance of this discussion and the hypothesis of the applicability of these peptides in the prevention and/or therapy of inflammation present in overweight and obesity, this article aims to conduct a review narratively and systematically organized concerning the mechanisms of action of hydrolyzed proteins and peptides of plant source in inflammation affected by adipose tissue excess. For the inclusion of articles, searches were performed in databases using descriptors and terms that include obesity, inflammation, proteins and peptides of plant origin.

## 2. Hydrolyzed Proteins, Peptide Pool and Their Respective Anti-Inflammatory Mechanisms of Action

The hydrolysis process on proteins can generate different peptides depending on the enzyme used in the reaction, the enzymatic combination and the reaction time. All these factors alter the developed products, which should be well evaluated. Furthermore, there is no way to define which peptide specifically had a particular effect in a peptide pool or even if the action was due to the set of peptides contained in the hydrolysis pool [22]. Considering these observations, the mechanisms of the pool of hydrolyzed proteins from some plant sources stand out.

Soy (*Glycine max*) is a legume source of proteins, with high nutritional value, economically crucial in the world market and widely studied [23,24]. Martinez-Villaluenga et al. [25] observed that soy-derived peptides, from hydrolysis with Alcalase (SPH), acted by reducing lipid accumulation in 3T3-L adipocytes culture because it reduced the expression of lipoprotein lipase (*LPL*) and fatty acid synthase (*FAS*) genes. In addition, it decreased, in a mouse macrophage cell culture (RAW264.7) stimulated with lipopolysaccharide (LPS), the production of NO, prostaglandin-endoperoxide synthase 2 (PGE2), the protein expression of nitric oxide synthase (iNOS) and cyclooxygenase-2 (COX-2).

Yi et al. [24] investigated the effect of soy protein-derived peptides (SBP)—acquired commercially—on inflammation in raw 264.7 cell culture. SBP reduced the concentrations and mRNA expression of the cytokines TNF-α, IL-1β and IL-6, and the protein expression of TNF-α occurred dose-dependently. In addition, BPS minimized the expression of both lymphocytic antigen 96 mRNA (*LY96*) and the toll 4 receptor protein (TRL4) dose-dependently, suppressing the phosphorylation of the α-NF-κB inhibitor (IκBα), suggesting a reduction in NF-κB activation. The phosphorylation of NF-κB, P-85, P-AKT and P-IKKe was evaluated, and the higher doses of SPB reduced the expression of such proteins [24].

Oseguera-Toledo et al. [26] analyzed the in vitro antioxidant capacity of the common bean protein (*Phaseolus vulgaris* L.) of two varieties, Negro 8025 (N) and Pinto Durango, (P) and its action in raw macrophage culture 264.7. Both varieties showed increased antioxidant capacity as the hydrolysis time with Alcalase occurred (with digestion product ranging between 43 and 50 kDa and between 20 and 25 kDa), favoring the reduction of oxidative stress. Therefore, the peptide pool was evaluated on macrophage culture compared with the induction of inflammation by LPS. Macrophages treated with common bean hydrolysate induced a lower production of NO and iNOS. In addition, a potent reduction in PGE2 production and an intense reduction in COX-2 protein expression after 120 and 180 min of enzymatic hydrolysis was observed. The pool of hydrolyzed peptides significantly reduced both the activation of the nuclear kappa B factor (NF-κB) and the nuclear translocation of subunits p65 and p50 [26].

The authors then tried to characterize these peptides, performing theoretical enzymatic digestion (by the Expert Protein Analysis System—ExPaSy) with the enzymes used in the experiment. The peptides found presented molecular masses, similar to those found by electrophoresis in polyacrylamide gel with sodium-dodecylsulfate polyacrylamide gel electrophoresis (SDS-PAGE). According to theoretical digestion, the peptides RSGSAILVLV, SFATSLREE, DNPIFSDHQ, SGSYFVDGHH and NEGEAH were candidates for synthesis in after-studies, especially those containing residues of asparagine (Asn) [26].

Another vegetable protein of the legume family, extracted from lupine (*Lupinus angustifolius* L.), was hydrolyzed (LPHs) with Izyme AL and/or Alcalase 2.4 L and evaluated in vitro for its inhibitory potential of enzymes involved in the inflammatory process [27]. The Izyme enzyme has trypsin-like activity, and Alcalase is a non-specific endoprotease. LPHs hydrolyzed with Izyme AL for 60 min inhibited phospholipase A_2_ (PLA_2_) [27]. PLA_2_ is an enzyme that catalyzes the hydrolysis of phospholipids, releasing phosphatidylcholine and phosphatidylcholine, which promotes synthesis of arachidonate—the precursor for the synthesis of eicosanoids: prostaglandin, leukotriene and thromboxane. Therefore, the mechanism of LPHs in inhibiting PLA_2_ is directly related to the reduction of lipid peroxidation. This metabolic pathway is the target of several steroidal anti-inflammatory drugs, such as prednisone and prednisolone [28].

Further, all the hydrolyzed LPHs tested inhibited COX-2 by 60% [27] without the distinction of hydrolysis reaction time. Regarding thrombin inhibition, this did not exceed 40%, and inhibition of the enzyme transglutaminase (TG) occurred when there was combined hydrolysis of Izyme AL and Alcalase. The authors reported a difference between the peptides generated according to the time and enzyme used, which may have diverse potentials under the inhibition of the enzymes involved in the inflammatory cascade tested [27].

In another study with digested LPHs also using Izyme AL and Alcalase 2.4, evaluating their effects on monocyte cell culture (THP-1, ATCC^®^ no. TIB-202™), there was a reduction in the expression of mRNA of inflammatory cytokines, *TNF*, *IL-6* and *IL-β*, markers of the M1-like phenotype of macrophages. Although there was no difference in the expression of mRNA of the *IL-10*, there was an increase in the expression of the anti-inflammatory marker gene of chemokine (*C-C motif*) *ligand 18* (*CCL18*), which is a marker of the phenotype M2. NO production halved and CC chemokine receptor type 2 (CCR2) mRNA expression and the THP-1 macrophage migration index statistically decreased, although digested LPHs did not decrease monocyte chemoattractant protein-1 (MCP-1, monocyte chemoattractant protein-1). Thus, according to the authors, the pool of peptides derived from lupine-derived protein hydrolysis favored macrophage polarization for type M2, which has an anti-inflammatory character [29].

More recently, LPHs hydrolyzed only with Alcalase 2.4. were evaluated in an ex vivo model in human peripheral blood mononuclear cells (PBMCs) of healthy donors. Cruz-Chamorro et al. [30] reported the activity of LPHs in PBMCs as immunomodulators and antioxidants via superoxide dismutase (SOD) and catalase (CAT) enzyme activities. First, no effects of LPHs on cell viability were detected. Therefore, the immunological effect of this peptide pool was evaluated, and the tested doses (0.5 and 0.75 mg/mL) significantly reduced the concentrations of pro-inflammatory Th1 cytokines: IL-2, IL-12, IFN-γ and TNF, and decreased the concentrations of IL-17, IL-9 and IL-13. However, there was a reduction in the production of IL-10, which is an anti-inflammatory cytokine. LPHs were able to increase the expression of mRNA and the enzymatic activity of *SOD* and *CAT,* not altering the mRNA expression of glutathione peroxidase (GPx) and glutathione reductase (GR) [30]. SOD and CAT are essential antioxidant enzymes in the redox potential, balancing oxidative stress (OE), chronically unbalanced in the context of obesity, causing a vicious cycle between mitochondrial dysfunction and increased production of reactive oxygen species (ROS). Moreover, the increase in OE has a strong relationship with visceral fat accumulation and metabolic syndrome (MS) [31,32].

In the study by Cruz-Chamorro et al. [30], LPHs also presented a polarizing character for the Th2 protective phenotype, considering a system based on unimmortalized human cells, and increased the total antioxidant capacity (TAC). In addition, the need to characterize each peptide generated from hydrolysis was also discussed in the study.

With the same objective of evaluating the immunomodulatory effect and antioxidant capacity of hydrolyzed wheat gluten protein (WGPH) with Alcalase 2.4 L, Cruz-Chamorro et al. [33] tested WGPHs in PBMCs of healthy donors. Even though wheat is one of the main food sources for the world population, it is also a problem for many individuals with celiac disease, which also has a strong inflammatory aspect [33,34]. Thus, the study suggests that the hydrolysis process can help reduce wheat antigenicity [33].

In the ex vivo model, WGPHs decreased the production of IFN-γ, IL-17 as well as reduced the expression of mRNA of *iNOS* and the production of NO. However, it also minimized the production of IL-10 and did not affect IL-4 and significantly increased the expression of *IL-10* mRNA. Among the antioxidant enzymes, CAT, GPx, SOD and GR, only the expression of GR mRNA was significantly increased, as well as increased antioxidant parameters: TAC, oxygen radical absorbance capacity (ORAC), ferric reducing antioxidant power (FRAP) and equivalent antioxidant capacity of trolox (TEAC) and antioxidant capacity equivalent to trolox (TEAC). Finally, the authors discuss that WGPHs significantly increased the molar ratios of Th2/Th1 and Th2/Th17 cytokines, promoting a shift in the pro-inflammatory response towards an anti-inflammatory microenvironment [33].

According to the studies addressed, the pool of peptides derived from direct protein hydrolysis from the source or even acquired commercially presented promising results as anti-inflammatory agents. Most of them acted by reducing inflammatory cytokines, favoring the polarization of M2 macrophages and showing a significant antioxidant response (Table 1).

## 3. Anti-Inflammatory Mechanisms of Action of Purified Peptides Obtained from Hydrolyzed Proteins of Plant Origin

Bioactive peptides are oligopeptides usually inactive or latent within intact proteins, only released through enzymatic hydrolysis, fermentation or physiological digestion, thus becoming active to perform important functions according to their structure and composition [18].

Due to the various potential therapeutic effects of peptides—such as anticoagulant, chelating effect, anti-obesity, antioxidant, antimicrobial, anti-diabetic [19,20,35]—there is an interest in discovering their mechanism of action and safety. In the search for studies with peptides obtained from hydrolyzed proteins of plant source, those with experimental models in cell cultures to verify their anti-inflammatory activity were found.

According to Millán-Linares et al. [36], the peptide derived from the protein isolated from the lupine of the legume *Lupinus angustifolius* L., whose amino acid sequence is GPETAFLR, with molecular mass <10 kDa, showed anti-inflammatory activity in a culture of THP-1 cells derived from macrophages. Octapeptide was able to reduce the protein expression of TNF, IL-1β, CCL2 and NO production, and further increased IL-10 cytokine.

More recently, Paz et al. [37] made progress in studies with the octapepetid GPETAFLR, analyzing it in a culture of monocytes derived from PBMCs of healthy donors. The effect was a reduction in classical monocytes (CD14^++^CD16^−^), which represent the main fraction among the types of monocytes, reduction in *CCR2* protein and mRNA expression, favoring the polarization for M1-like, and reduction in response to LPS, which secretes inflammatory cytokines [38]. Due to this characteristic, the authors also analyzed and verified that the peptide reduced the protein expression of CCR2, the secretion of TNF-α, IL-1β and IL-6 and the expression of the genes of these cytokines dose-dependently. In addition, GPETAFLR increased the secretion and gene expression of *IL-10.* Regarding the macrophage polarization assay, GPETAFLR reduced the mRNA expression of *CD80* and *CD64* and increased *CD200R* and C receptor of mannose type 1 (*MRC-1*) in M0; i.e., there was a decrease in the pro-inflammatory activity of the M1-like phenotype.

A soy tripeptide (*Glicine max*), whose sequence is FLV, was tested in macrophage and adipocytes culture by Kwak et al. [39]. It inhibited the secretion of TNF-α, MCP-1 and IL-6, and the anti-inflammatory mechanism was explained by the inhibition of TNF-α-induced activation of the C-Jun N-terminal kinase (JNK)/kinase IκB (IKK) signaling pathway and negative regulation of IB in adipocytes. In addition, adipocytes culture expressed a peptide carrier protein 2 (PepT2). This protein was responsible for transporting the FLV peptide to the intracellular medium, acting by deregulating the inflammatory signaling induced by TNF-α and favoring insulin sensitivity.

In another study, by Hashidume et al. [40], soy-derived peptides had insulin-like action in skeletal muscle cells (rat L6 and mouse C2C12). The studied peptides were leginsulin 1_36 and 1_37; the difference between them is a GLY residue in the C-terminal. Both peptides activated protein kinase B (Akt) and increased the translocation of glucose transporter 4 (GLUT4), suggesting insulin-like activity. The residues I25, F28, V29, F31 and I33 on the peptide surface were crucial for affinity with the storage protein of soybean seed basic 7 S globulin protein (Bg), which has a structure similar to the insulin receptor [41].

Peptides derived from sunflower seed (*Helianthus annuus* L.) also present bioactive properties [42]. Four small and non-polar peptides were obtained, sequenced (YFVP, SGRDP, MVWGP and TGSYTEGWS) and tested in THP-1 cells (ATCC^®^ TIB-202™). All were able to inhibit the activation of NF-κB by IL-1, and the last two peptides had an increased effect on this inhibition. In addition, the CD14 surface marker induction also increased. However, only MVWGP and TGSYTEGWS increased the protein expression of CD86. The peptide containing the Met residue presented the most promising anti-inflammatory and immunomodulatory effect among the four studied peptides of sunflower seed. According to the authors, the explanation is not completely clear [42]. However, the Met is a precursor of S-adenosil-L-methionine (SAMe or AdoMet), which is an enzyme with potential alkylating, a methyl donor, actively participating in numerous biochemical processes, such as a glutathione precursor (GSH), which acts in the detoxification process [43,44].

Among the three peptides obtained from corn protein (zein), the one with Met was also highlighted in the study by Liang et al. [45]. In this research, the three synthesized peptides (PPYLSP, IIGGAL and FLPPVTSMG) were tested for their anti-inflammatory capacity in endothelial cell culture (EA.hy926, CRL-2922™) and monoblasts (U937, CRL-1593.2™). All the peptides inhibited the protein expression of intercellular adhesion molecule-1 (ICAM-1) and vascular cell adhesion molecule-1 (VCAM-1) induced by TNF-α and reduced the adhesion of monocytes to endothelial cells, with the peptide FLPPVTSMG presenting the best results. They also reduced the formation of superoxide, which is directly linked to oxidative stress, minimized the expression of TNF-α receptor 1 (TNFR-1)—related to inflammation and apoptosis—but not of receptor 2 TNF-α (TNFR-2), which has a primary function of cellular survival. The anti-inflammatory action of these peptides seemed to be independent of the degradation of IκBα and IκBβ due to the low effect in the experiment. However, they inhibited the phosphorylation of p65, one of the dimers involved with the NF-κB pathway, thus inhibiting inflammatory signaling [45].

By analyzing the mechanism of action of plant peptides in inflammation, it is possible to infer that they act by reducing the release of inflammatory cytokines. This action favors a breakdown of positive feedback between fat accumulation and inflammatory cytokines, inhibiting NF-κB and reducing the expression of the TNF-α receptor (Table 2).

## 4. Metabolic Pathways of Inflammation in Obesity: Potential Therapeutic Targets for Hydrolyzed Proteins and Plant Peptides

It is classic information that the white adipose tissue (WAT) is an endocrine organ, especially after discovering leptin. WAT has a primary function in energy metabolism through a metabolic network involving the central nervous system (CNS). In addition, it is responsible for secreting several adipokines and cytokines that have autocrine, paracrine and endocrine action—involving immune function and fundamental for the metabolism of sex steroids and glucocorticoids. Due to these numerous functions, excess WAT causes a series of metabolic disorders [46].

These disorders favor the increase in signaling for adipogenesis, causing both hypertrophy and hyperplasia of adipocytic cells, increased fatty acids, and circulating free inflammatory cytokines, as well as changes in the signaling of hormones that regulate the hunger–satiety axis. All these changes are complex and triggered by signaling pathways, genes and transcription factors [12,47]. Thus, it is essential to unveil potential molecular targets for the action of anti-inflammatory therapeutic agents.

In a study with adipose tissue cells extracted from rats and cells donated from patients who underwent bariatric surgery, Zhou et al. [48] described an association between low-grade chronic inflammation, caused by increased fat accumulation, and energy mitochondrial metabolism changes. The association was explained by increasing secretion and signaling of inflammatory cytokine IL-1β, which interacts with the interleukin-1 receptor (IL-1R), recruiting the IL-1R-associated kinase 2 (IRAK) and myeloid differentiation primary response 88 (MyD88). With this activation, a cascade of protein interactions is triggered, affecting the morphology of the crests of the mitochondrial membrane, causing suppression of the electron carrier chain. This mechanism also impairs thermogenesis in brown adipose tissue (BAT). The outcome was the reduction in energy expenditure with the consequent increase in WAT [9,48].

According to Zhou et al. [48], the discovery of this unconventional metabolic pathway, IL-1R-IRAK2, favors the search for therapeutic agents that have binding affinity with IRAK2 protein to minimize the inflammatory response induced by the increase in IL-1β affected by excess adipose tissue. Two pools of hydrolysis of plant protein, SBP [24], LPHs [29], FLV [39] and GPETAFLR [36,37], reduced the protein expression of IL-1β, characterizing these molecules as potential anti-inflammatory agents acting in this signaling pathway.

This mechanism still unfolds, as reported by Yu et al. [49], in a study with macrophages derived from the bone marrow of rats with systemic inflammation induced by a high-fat diet and human low-density lipoprotein (LDL). The protein MyD88 interacted with TLRs, triggering an inflammatory response due to increased macrophage recruitment to the adipose tissue and polarization to the M1-like phenotype. As a result, insulin resistance increased. Among the studies included in the present review, SBP [24] acted to reduce the protein expression of TLR4, a beneficial action to attenuate the inflammatory response.

Furthermore, the increase in IL-1β also participates in the activation and positive reinforcement of the metabolic pathway of NF-κB, classically known as the transcription factor related to the production of pro-inflammatory components. Mollaei et al. [50] presented in their review a cascade of activation of the NF-κB transcription pathway by LPS, in which, as soon as there is binding between LPS and the LPS binding protein (LBP), it triggers the following response: (1) phosphorylation of IκBα, (2) degradation of IκBα, (3) activation of transcription factors that are taken to DNA and (4) signaling for the synthesis and release of chemokines—such as macrophage inflammatory protein-1 (MIP-1), MCP-1, also known as CCL2, and interferon (IFN)-γ inducible protein 10 (IP-10)—and the release of enzymes—COX-2 and iNOS—and cytokines—such as IL-1β, TNF-α and IL-6.

According to a study with adipocytes derived from ear mesenchymal stem cells (EMSC Ad) and dendritic bone marrow cells (BMDCs) of wild rats (C57Bl/6 J), knockout for TRL4 (*Tlr4^−/−^*), fed ad libitum with feed containing 4.5% fat, this whole cascade was also triggered in the presence of saturated free fatty acids (AGLS) when binding to TLR4 and TLR2 [51].

Another study [52] also analyzed the action of free saturated fatty acid (FFA) on adipose tissue of wild rats (C57Bl/6J and *Tlr4^−/−^*), and in obese rats knocked out for My88 (*Myd88^−/−^*) and for TIR domain-containing adapter protein-inducing interferon-β (TRIF) (*Trif^−/−^*), comparing animals with high-fat diet-induced obesity (HFD) (60% of calories from lipids) and groups with diet containing 4.5% lipids. The TRL4 and the HFD conditions were fundamental for the increase in the recruitment of adipose tissue macrophages (ATMs) CD11c^+^. The presence of MyD88 and TRIF favor this recruitment, accentuating inflammation and culminating in macrophage polarization.

It is well documented that TRL4 is expressed in innate immune cells and other cell types, such as enterocytes and adipocytes, and both the presence of LPS and fatty acids can stimulate the inflammatory response via TRL4 [53,54]. Therefore, this pathway is essential in studying bioactives with anti-inflammatory potential. In the study by Martinez-Villaluenga et al. [25], the pool of peptides of hydrolyzed soybean protein (SPH) reduced free fatty acids, evidencing a possible consistent therapeutic target in inflammation.

Moreover, in relation to the NF-κB pathway, the hydrolyzed proteins of beans from the black 8025 (N), Pinto Durango (P) [26] and SBP varieties [24]; and peptides extracted from corn (zein) [45] and sunflower [42] were able to reduce NF-κB activity, consequently decreasing the expression of inflammatory cytokines, COX-2 and iNOS.

The signaling cascade of TNF-α is per si stimulated during obesity and hyperglycemia, causing an activation response of NF-κB that increases iNOS, which inhibits the substrate of the insulin receptor substrate 1 (IRS-1), and relates to the increase in OE, hindering the functioning of mitochondria and causing a reduction in β-oxidation [55,56,57,58]. This signaling pathway is of great importance in studies of obesity and inflammation because the peptides of zein [45] reduced the expression of the TNF-α receptor. In addition, the octapeptide of lupine [36], FLV [39], the hydrolysis pool of SBP [24] and LPHs [29] reduced the cytokine expression, aiding in reducing the entire cascade related to the TNF-α signal.

Another signaling pathway involved in inflammation is JAK/STAT (Janus kinase and transducer signal and activator of transcription), which regulates the synthesis of inflammatory cytokines, hormones and growth factors by rapid transduction of intracellular signal [59]. The signaling cascade occurs through the communication between cytokines and class I and II receptors in the cell membrane, causing activation of JAKs [60], which phosphorylate and allow the fitting with STAT proteins, which are also phosphorylated. Thus, STAT multidimers can promote epigenetic changes by regulating gene transcription in the nucleus [61,62]. Considering the JAK/STAT pathway, most hydrolyzed proteins and peptides acted by minimizing this activation cascade. These potential anti-inflammatory agents decrease the protein expression of several cytokines that positively feed the pathway.

JAK/STAT signaling is determinant in cellular communication because several types of JAK and STAT guarantee specificity [61]. The leptin/JAK2/STAT3 pathway is positively refueled by IL-6, and this activation profile is fundamental for the development of insulin resistance and inflammation-related glucose intolerance. STAT3 is a facilitating agent of adipogenesis through the peroxisome proliferator-activated receptor (PPAR-γ) [54,63].

In obesity, there is also an increase in the synthesis and release of IL-6, which acts on STAT3, favoring the activation of the suppressor of cytokine signaling 3 (SOCS3) [63,64]. In the study by Shi et al. [64] in 3T3-L1 adipose tissue cells, SOCS3 inhibited IRS-1, thus inhibiting the PIK3-AKT signaling pathway, causing insulin resistance (IR).

Regarding IR, it is still discussed that the recruitment or replication of M1 in the pancreas, in the condition of excess adipose tissue, promotes hyperplasia and dysfunction of β-cells, therefore impairing insulin secretion. Thus, the metabolic pathways related to glucose metabolism are potential therapeutic targets [65].

Furthermore, among the vegetable peptides, the peptide Leg (Leg_1_37 and Leg_1_36) [40] was able to increase the activity of the phosphatidylinositol 3-kinase (PI3K) and AKT pathways, favoring GLUT-4 translocation and assisting in the reduction of IR, minimizing IR and improving inflammation.

STAT3 also regulates hematopoiesis and signaling for immune cells, as is the case with T helper 17 (Th17) lymphocytes. The differentiation process of Th17 occurs in response to STAT3 to low concentration signaling of IL-21, IL-6 and IL-23, in addition to beta growth transformation factor (TGF-β) and IL-1, by means other than STAT3 [66]. Th17 cells secrete IL-17, which contributes to an increased macrophage-mediated pro-inflammatory response inducing secretion of IL-1β, IL-6 and TNF-α [6,67]. Therefore, due to JAK/STAT dysregulation not only in obesity but also in T2DM and MS, it is a potential therapeutic target for such metabolic disorders [68].

**Figure 1:**
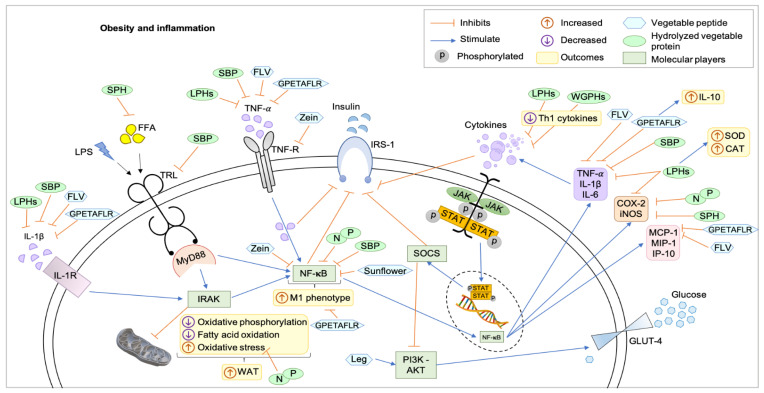
Mechanisms of action of hydrolyzed proteins and plant peptides on obesity and inflammation in a hypothetical eukaryotic cell. All hydrolyzed proteins and peptides acted to reduce inflammation through metabolic pathways that communicate and are responsible for the positive feedback cascade for the accumulation of more adipose tissue and subclinical inflammation, as well as some compounds acted by feeding detoxification and detoxification pathways. reduction of oxidative stress. CAT: catalase, COX-2: cyclooxygenase-2, FFA: free fatty acid, FLV: soy peptide Phe–Leu–Val, GLUT4: glucose transporter type 4, GPETAFLR: peptide from *Lupinus angustifolius* L., IL-10: interleukins-10, IL-6: interleukins-6, IL1-β: interleukins-1β, IL1-R: interleukins-1-receptor, iNOS: inducible nitric oxide synthase, IP-10: interferon-inducible protein 10, IRAK: IL-1R-associated kinase 2, IRS-1: insulin receptor substrate 1, JAK-STAT: Janus kinase—signal transducer and activator of transcription, Leg: Leginsulin, LPHs: lupine protein hydrolysates, LPS: lipopolysaccharides, M1: M1-like macrophage, MCP-1: monocyte chemoattractant protein-1, MIP-1: macrophage inflammatory protein-1, MyD88: myeloid differentiation primary response 88, N and P: protein hydrolysates of the common bean (*Phaseolus vulgaris* L.) varieties Negro 8025 and Pinto Durango, NFκB: factor nuclear kappa B, PI3K-AKT: phosphatidylinositol 3-kinase/protein kinase B, SBP: soybean protein-derived peptides, SOCS: suppressor of cytokine signaling, SOD: superoxide dismutase, SPH: soy protein peptic hydrolysate, sunflower: peptides from sunflower protein hydrolysate, Th1: T helper 1, TNF-α: tumor necrosis factor-α, TNF-R: tumor necrosis factor-receptor, TRL: toll-like receptor, WAT: white adipose tissue, WGPHs: wheat gluten protein hydrolysates, zein: peptides for zein hydrolysate.

Figure 1 shows updates on the metabolic pathways of obesity inflammation and the potential therapeutic targets of hydrolyzed proteins and purified peptides in a hypothetical eukaryotic cell. Studies have their limitations, either by the experimental model used or by the study design, which do not cancel the possibility of effects by other mechanisms. Among the 13 included studies using peptide pools, one study addressed the improvement in the profile of enzymes related to hepatic detoxification [30], seven analyzed the reduction in oxidative stress and increased antioxidant activity [25,26,29,30,33,36,37], five studies found reduction in NF-κB [24,26,39,42,45], two verified glucose uptake pathways [39,40] and all 13 analyzed inflammatory cytokines, mainly TNF-α and IL-1β, and improvements in immunomodulation [24,25,26,27,29,30,33,36,37,39,40,42,45].

Therefore, the mechanisms of action were the reduction in inflammatory cytokines and enzymes involved with the inflammatory process; inhibition of NF-κB; reduction in OE; stimulation of the PI3K-AKT pathway and enzymes related to detoxification.

All the studies discussed in this review were conducted in cell cultures except for one, which was an in vitro enzymatic assay. This shows the need to deepen the studies through research in animals and in clinical trials. However, a determining limitation, which contributes to the difficulty in making original articles in an animal model with bioactive peptides, is the high financial cost [69]. In addition, there is a long methodological process to purify a plant [70], hydrolyze in sufficient quantity to test in animals and synthesize and identify peptides so that the mechanism of action involved is established.

Bioinformatics can help predict molecular targets that have a potential affinity for compounds or molecules of interest and identify new biomarkers for prognosis and diagnosis, identifying and analyzing differentially expressed genes (DEGs) in various diseases [71,72]. Moreover, this area can also contribute to the search for alternative drugs for new diseases, providing an extremely useful tool to accelerate urgent studies for the selection of these drugs, understanding and possibly helping in the treatment of new diseases, whether chronic, such as obesity, or acute and emerging, as in the case of coronavirus disease-19 (COVID-19)—since bioinformatics was and still is an essential method to perform sequencing data analysis, pandemic tracking and analysis of pandemic containment measures [73,74,75].

## 5. Conclusions

The present review showed that hydrolyzed proteins and peptides of plant sources are promising molecules both as anti-inflammatory agents and with immunomodulatory function, presenting mechanisms of action in several metabolic pathways that correlate. According to the evaluated studies, it is possible to infer that this anti-inflammatory activity is directly related to the polarization of macrophages to the M2 phenotype. However, there is a need for more understanding of these molecules of natural origin using other types of studies, extrapolating the limitations of research that use cell and ex vivo culture. Thus, the development of bioinformatics tools for such analysis is supported to predict molecular targets that are determinant in overweight and obesity and other emerging diseases, adding to different research strategies that safely promote potential new bioactive molecules.

## Figures and Tables

**Table 1:** Anti-inflammatory and immunomodulatory effects of hydrolyzed vegetable proteins reported in the literature.

Vegetable Protein	Cell Culture/Enzyme Assay	Enzymes Used in Hydrolysis	Outcomes
Hydrolyzed soy protein (SPH) [25]	Macrophage cells, RAW 264.7	Alcalase 2.4 L	↓ Expression of *LPL* and *FAS* genes ↓ Production of NO and iNOS ↓ Production of PGE2 and COX-2
Common beans (*Phaseolus vulgaris* L.), varieties Negro 8025 (N) and Pinto Durango (P) [26]	Macrophage cells, RAW 264.7	Alcalase 2.4 L	↓ NO production ↓ Production PGE_2_ and COX-2 ↓ Nuclear translocation of NF-κB subunits, p50 and p65 ↓ NF-κB activation
Seeds of lupine (*Lupinus angustifolius* L.) (LPH) [27]	In vitro inhibition of enzymes	Izyme AL and Alcalase 2.4 L	↓ PLA_2_ and thrombin activity when LPH hydrolyzed with Izyme ↓ Transglutaminase activity when LPH hydrolyzed with Izyme and Alcalase ⊗ COX-2 activity by LPH without distinction of time or type of hydrolysis
Seeds of lupine (*Lupinus angustifolius* L.) (LPH) [29]	THP-1 monocytes (human acute monocytic leukemia) (ATCC^®^-TIB-202™)	Izyme AL and Alcalase 2.4 L	↑ CCL18 expression ↓ Expression of TNF, IL-6 and IL-1β ↓ NO production ↓ CCR2 expression
Seeds of lupine (*Lupinus angustifolius* L.) (LPH) [30]	PBMCs of 53 healthy adult donors	Alcalase 2.4 L	↓ Expression of IL-2, IL-12, IFN-γ, TNF, IL-17, IL-9 and IL-13 ↑ Gene expression and SOD and CAT activity ↑ TAC ↑ Protection against cell death mediated by oxidative stress induced by H_2_O_2_
Wheat gluten protein (WGPHs) [33]	PBMCs of 39 healthy adult donors	Alcalase 2.4 L	↓ Production of IFN-γ and IL-17 ↓ *iNOS* gene expression and NO production ↓ Cell proliferation of Th1 and Th17 ↑ *GR* gene expression ↑ TAC, ORAC, FRAP e TEAC ↑ Th2/Th1 and Th2/Th17 Balance Sheet
Pool of soy-derived peptides (*Glycine max*) (SBP) [24]	Macrophage cells, RAW 264.7	The peptide pool was acquired commercially	↓ Gene expression and activity of TNF-α, IL-1β and IL-6 ↓ *LY96* gene expression ↓ TLR4 expression ↓ Phosphorylation of IκBα ↓ P-85, P-AKT and P-IKKe expression

↓ Reduces, ↑ Increases, ⊗ Inhibits.

**Table 2:** Anti-inflammatory and immunomodulatory effects of plant peptides reported in the literature.

Vegetable Protein	Cell Culture	Peptide(s)	Outcomes
Seeds of lupine (*Lupinus angustifolius* L.) (LPH) [36]	Thp-1 monocytes (acute monocytic human leukemia) (ATCC^®^-TIB-202™).	GPETAFLR	↓ Expression of TNF, IL-1β and CCL2. ↑ IL-10 expression. ↓ NO production There was no statistical difference in the expression of the *IL-6* and *CCL18* gene
Soy (*Glicine max*) [39]	Macrophage cells, RAW 264.7 and adipocytes 3T3-L1	FLV	⊗ Release of TNF-α, MCP-1, IL-1β and IL-6 ↑ Increasing IL-10 production and gene expression ⊗ Signaling molecules JNK and IKK ⊗ IκBα degradation ↑ Insulin response and glucose uptake FlV can be transported to adipocytes by PepT2 and block TNF-α-induced inflammatory signaling.
Soja (*Glycine max*)–variant leginsulin (Leg_1_37 and Leg_1_36) [40]	Rat L6 and mouse C2C12 skeletal muscle cells.	ADCNGACSPFEVPPCRSRDCRCVPIGLFVGFCIHPTG and ADCNGACSPFEVPPCRSRDCRCVPIGLFVGFCIHPT	◊ Akt ↑ GLUT4 translocations to the plasma membrane. Leg1_37 showed insulin-like activity Amino acid residues I25, F28, V29, F31 and I33are critical for affinity with Bg.
Seeds of lupine (*Lupinus angustifolius* L.) [37]	PBMCs from healthy adult donors	GPETAFLR	↓ Population of classical monocytes (CD14^++^CD16^−^). ↓ Expression of CCR2 and CCL2. ↑ Production and expression of *IL-10* genes. ↓ Gene expression and production of IL-1β, IL-6 and TNF-α. ↓ Pro-inflammatory activity of the M1-like phenotype.
Sunflower seed (*Helianthus annuus* L.) [42]	Thp-1 monocytes (acute monocytic human leukemia) (ATCC^®^-TIB-202™)	YFVP, SGRDP, MVWGP and TGSYTEGWS	Small, non-polar peptides ⊗ NF-κB activated by IL-1β ↓ Expression of CD14 and CD86 Peptides with Met had superior immunomodulatory effect
Corn (Zein) [45]	Endothelial cells, EA.hy926 (CRL-2922™), and human monoblast cell lineage, U937 (ATCC^®^ CRL-1593.2™)	PPYLSP, IIGGAL and FLPPVTSMG.	↓ Expression of ICAM-1 and VCAM-1 induced by TNF-α. ↓ Monocyte stems from EA.hy926 cells. The peptide with Met had the best result. ↓ TNF-α-induced superoxide formation in various degrees in EA.hy926 cells. ↓ Expression of TNFR1 in EA.hy926 cells. Little effect on the expression of IκBα and IκBβ induced by TNF-α ↓ Phosphorylation of p65: dummer involved in the NF-κB pathway.

Legend: ↓ Reduces, ↑ Increases, ⊗ Inhibits, ◊ Active.

## Data Availability

Not applicable.
